# Improving Long-Term Outcomes After Extracorporeal Membrane Oxygenation: From Observational Follow-Up Programs Toward Risk Stratification

**DOI:** 10.3389/fped.2018.00177

**Published:** 2018-06-26

**Authors:** Hanneke IJsselstijn, Maayke Hunfeld, Raisa M. Schiller, Robert J. Houmes, Aparna Hoskote, Dick Tibboel, Arno F. J. van Heijst

**Affiliations:** ^1^Division of Pediatric Intensive Care, Erasmus Medical Center, Sophia Children's Hospital, Rotterdam, Netherlands; ^2^Department of Pediatric Surgery, Erasmus Medical Center, Sophia Children's Hospital, Rotterdam, Netherlands; ^3^Department of Cardiac Intensive Care, Great Ormond Street Institute of Child Health, University College London and Great Ormond Street Hospital for Children, London, United Kingdom; ^4^Department of Neonatology, Amalia Children's Hospital, Radboud University Medical Center, Nijmegen, Netherlands

**Keywords:** extracorporeal membrane oxygenation, long-term outcomes, neurodevelopment, neuromonitoring, follow-up

## Abstract

Since the introduction of extracorporeal membrane oxygenation (ECMO), more neonates and children with cardiorespiratory failure survive. Interest has therefore shifted from reduction of mortality toward evaluation of long-term outcomes and prevention of morbidity. This review addresses the changes in ECMO population and the ECMO-treatment that may affect long-term outcomes, the diagnostic modalities to evaluate neurological morbidities and their contributions to prognostication of long-term outcomes. Most follow-up data have only become available from observational follow-up programs in neonatal ECMO-survivors. The main topics are discussed in this review. Recommendations for long-term follow up depend on the presence of neurological comorbidity, the nature and extent of the underlying disease, and the indication for ECMO. Follow up should preferably be offered as standard of care, and in an interdisciplinary, structured and standardized way. This permits evaluation of outcome data and effect of interventions. We propose a standardized approach and recommend that multiple domains should be evaluated during long-term follow up of neonates and children who needed extracorporeal life support.

## Introduction

Since the Extracorporeal Life Support Organization (ELSO) registry was launched in 1989, data of more than 35,000 neonates and 20,000 children treated with extracorporeal membrane oxygenation (ECMO) have been registered (1). Neonates were treated with ECMO mainly for respiratory failure; older children more frequently for cardio-pulmonary failure (1).

The indications and starting criteria for ECMO in neonates are well defined. There is no evidence based criteria for older children, likely due to the diverse etiology of the cardiopulmonary failure in this age group (2, 3). As cumulative experience with ECMO has increased, more and more complex patients with co-morbidities, who previously would not have qualified, are being referred for ECMO support (4). There is evidence for ECMO as a treatment modality for respiratory failure in neonates (5) and adults (6), but such evidence is lacking for the pediatric age range. Randomized pediatric trials addressing this issue have not been published. Uniform criteria, such as the oxygenation index in neonates and the Murray score of uncompensated hypercapnia with pH < 7.20 in adults, are not available for the pediatric age range. Recently, Barbaro and coworkers reported that in-hospital mortality and 90-day ventilator-free, hospital-free and PICU-free days did not differ between ECMO-supported children and non-ECMO supported controls in the RESTORE (Randomized Evaluation of Sedation Titration for Respiratory failure) clinical trial (7). The analysis was based on individual case matching and propensity score matching (7). A randomized controlled trial evaluating the real benefit of pediatric ECMO is still relevant, However, based on the study of Barbaro and coworkers (7), Carpenter and Kinsella calculated that more than 1,200 children per group should be included to show a significant difference in the mortality rate (8). The currently available guidelines for pediatric ECMO on the ELSO website clearly state that specific cut-offs at which ECMO should be offered or withheld have not been firmly established and therefore should be considered on a case-by-case basis. Important absolute contraindications include lethal chromosomal disorders and severe neurological compromise (3).

Of the ELSO-registered neonates, 75% were treated with ECMO for severe respiratory failure and overall survival to transfer or discharge in this group was 72% (1). Thus, seeing that an increasing number of critically ill neonates—with a high risk of mortality in the pre-ECMO era—survive, attention should be directed to long-term morbidity.

The long-term outcome of ECMO patients is very diverse due to the variety in underlying disease and additional comorbidities (9). Most follow-up studies in neonates and children who survived ECMO treatment have been cross-sectional, monodisciplinary, and in small study populations. The past few years have seen a shift toward long-term multidisciplinary evaluations, mainly following neonatal ECMO.

This review addresses both medical and neurodevelopmental outcomes after ECMO-treatment, as well as changes in ECMO programs that might potentially influence long-term outcomes. Recommendations will be made for follow up of these children beyond infancy and childhood.

## Changes in ECMO programs that might affect long-term outcomes

ECMO programs worldwide have been subject to several changes the last two decades. We distinguish two types of changes: First, changes in indications that may lead to a different population of survivors; and second, changes in techniques, devices and medical management that may affect outcomes.

### Changes in indications

Historically, many ECMO programs first focused on newborns with severe respiratory failure due to meconium aspiration syndrome (MAS), pneumonia/sepsis, congenital diaphragmatic hernia (CDH) or idiopathic persisting pulmonary hypertension of the newborn (PPHN). It appeared, however, that there was a high risk of complications and mortality for ECMO runs longer than 2–3 weeks (10). The indications in the newborn population have significantly changed since the introduction of high-frequency oscillatory ventilation, inhaled nitric oxide, intravenous sildenafil, and surfactant washout for MAS, among other modalities. Moreover, better obstetric care has lowered the prevalence of MAS and the resultant decrease in the rate of ECMO utilization in this population (11).

Indications for ECMO support in older children have broadened to include primary cardiac failure, post cardiac surgery patients and primary respiratory failure. Especially since the 2009 influenza H1N1 pandemic, ECMO is being applied more often and more in the older pediatric and adult populations with conditions that may require longer ECMO runs as a bridge to either recovery or transplantation (12). Despite the decline in survival with longer duration of ECMO, prolonged ECMO support in children appears reasonable unless multiorgan failure develops (4).

This change in policies implies that the previously excluded neonates and children with higher disease severity due to underlying disease ansd pre-existant morbidity can be treated with ECMO with a chance of survival.

Traditionally, cardiopulmonary resuscitation (CPR) was a contraindication for ECMO. A decade ago, however, ECMO was introduced as a rescue therapy during CPR (ECPR), and this resulted in higher survival rates and improved neurologic outcomes in comparison with standard CPR. Since then ECPR has been integrated in the Guidelines for Cardiopulmonary Resuscitation and Emergency Cardiovascular Care (13–15). Prospective randomized controlled studies that compare outcomes from ECPR with conventional CPR have not yet been reported; outcomes of ECPR have been described in observational studies and meta-analyses only. In the 2018 January report from the ELSO registry, survival to discharge following ECPR was 29% in 4,745 adults, 42% in 3,881 children and 40% in 1,694 neonates (1).

In adults treated with ECPR, survivors were discharged with favorable neurological outcome (Cerebral Performance Category 1 or 2) (16). The total arrest time proved to be the most important predictor for good neurological outcome and recovery of heart function in adult survivors of ECPR (17). In a study in children receiving ECPR, the Pediatric Cerebral Performance Category was 1 or 2 in 75% of survivors. Furthermore, there was a 52% reduction risk of CNS injury compared with the immediate pre-ECPR era (18). tThe clinical relevance of this score is limited, however, which will be discussed later. Studies evaluating long-term neuropsychological outcome following ECPR with formal developmental testing are lacking (19).

### Change in techniques, devices, and medical management during ECMO

Cardio-circulatory or respiratory failure, or a combination of both, is a dynamic process. Traditionally, venovenous (VV-ECMO) was provided for respiratory support and venoarterial (VA-ECMO) for circulatory support. For neonates, VA-ECMO was the preferred mode. Currently more hybrid forms of ECMO are used. To provide sufficient regional and systemic perfusion, cannulation for ECMO should be patient-tailored (20).

While a CNS injury rate as high as 20% has been reported in association with arterial cannulation, this rate is lower in association with VV-ECMO (21). A recent survey of the American Pediatric Surgical Association reported carotid artery repair after decannulation in 6.6% of all patients and in 10.7% of over 5 year old (22). The long-term effects of carotid cannulation and subsequent repair still remain unclear.

In adults, the stroke rate following arterial cannulation varied with the cannulation site: 5.9–22.7% for femoral, 6.2–10.6% for subclavian, 25% for carotid and 21.3% for aortic cannulation. In a single center study in 36 children with a body weight of at least 15 kg, the prevalence of neurologic injury was 25% following neck cannulation and 12% following femoral cannulation (23).

Traditional semi-occlusive roller pump ECMO systems with silicone membrane oxygenators have almost universally been replaced by centrifugal pumps and polymethylpentene diffusion membrane oxygenators. These newer systems are much easier to prime and require smaller priming volumes. Hemolysis has been reported as a complication of the use of centrifugal pumps in children (24). In other studies, however, pump type was not associated with bleeding, thrombosis, hemolysis, or mortality (25–27). The use of polymethylpentene oxygenators with less thrombogenic properties was associated with a trend toward a lower ICU mortality in patients. The question is whether the use of these next generation ECMO systems will result in better long-term outcome in children.

ECMO anticoagulation practices vary widely and have changed over the years. Although the ELSO has issued a guideline on the use of anticoagulation during ECMO (28), bleeding and clotting are still a major issue. The level of anticoagulation has to be judicially adjusted taking into account all complex factors that may affect such as the the severity of illness, underlying pathology and circuitry components. Bleeding occurred in 70% of a large cohort of pediatric ECMO patients, including intracranial hemorrhage in 16% (27). Neither hemolysis nor thrombotic events increased the risk of mortality (27). Thus, adequate monitoring of the coagulation system remains crucial and the level of anticoagulation might impact long-term outcome (29). Several high-volume ECMO centers from Canada, UK and the Netherlands have recently initiated a consortium—called the Phoenix consortium—to compare their standards of care and to prepare RCTs to optimize (anti)coagulant therapy. Although in a recent study in adult ECMO patients the benefit of bivalirudin versus heparin could not be shown, future newer types of anticoagulants may offer advantages (30).

## The prevalence of neurological morbidities and the role of diagnostic tools

Children supported on ECMO are at risk of neurological complications for a variety of reasons, ranging from poor pre-ECMO clinical state, severe underlying diagnosis, ECMO cannulation, systemic anticoagulation on ECMO, and other on-ECMO and post-ECMO complications. They should be carefully monitored, therefore, especially because they are likely to be sedated. Detailed clinical examination should be supplemented with cranial ultrasound scans (CUS), electro encephalography (EEG), near infrared spectroscopy (NIRS), transcranial Doppler, computer tomography (CT) brain and serum bio-markers. None of these modalities is perfect on its own, but in combination with good clinical examination and close bedside review by the ECMO specialists, acute neurological complications are likely to be identified and can be acted upon. The most important neurological complications are CNS hemorrhage and infarction, seizures, and, rarely, brain death (31–34).

### Hemorrhage and infarction

Between 2013 and 2017, CNS hemorrhage occurred in 12% of neonates undergoing respiratory ECMO, of whom 39%; CNS infarction occurred in 4%. of whom 33% survived (1). Corresponding figures for older children treated with ECMO for respiratory failure were 7% CNS hemorrhages with a 23% survival rate and 5% CNS infarctions with a 36% survival rate. Seizures occurred in 8 and 4% of neonates and older children, respectively, with survival rates of about 50 and 40% (1).

Cerebral lesions, including hemorrhage and ischemia, have been reported in 10–52% of neonates (35–37).

Some papers specifically described posterior fossa lesions in ECMO treated neonates (38, 39). It was speculated that ligation of the right jugular vein and cannulation caused obstruction of venous outflow and stasis, with the concomitant risk of rupture of the periventricular medullary veins (40).

As it is unclear whether all patients in the ELSO registry underwent neuro-imaging studies during and/or after ECMO, it is well possible that CNS complications are underestimated. A recent paper on serial CUS in 650 neonates treated with ECMO in the period 1989-2010 reported brain abnormalities in 17.3% (41). Primary hemorrhage was the most frequent abnormality (8.8%), located intraventricular in half of the cases. Stroke was detected in 5%, predominantly in the left hemisphere (70%). This was also the dominant hemisphere for lobar hemorrhages (2.2%; 89% left) (41). Seventy-five percent of the lesions were detected within the first 72 h of the ECMO run (41). Interestingly, the incidences of lesions had not changed over the years.

The ELSO recommends daily CUS in neonates after the start of ECMO (1). CUS is easily applied at low costs and with avoidance of radiation. Still, Biehl and coworkers suggested that daily CUS are cost-effective only during the first 3 days of ECMO. After 3 days, CUS is only indicated upon change in neurologic status or multi-organ failure, which events are both associated with the occurrence of late hemorrhages (42).

Moreover, CUS might also be used for Doppler studies. O'Brien and Hall demonstrated an increased risk for hemorrhages in children aged 2 days-18 years, as blood flow velocities measured in the middle cerebral artery were higher (43). Zamora and coworkers found that variations of more than 10% in the resistance index (i.e., peak systolic velocity-end diastolic velocity)/peak systolic velocity) of the anterior cerebral artery on any day of ECMO treatment were related with cerebrovascular complications (44).

However, small ischemic and hemorrhagic lesions may be difficult to recognize on CUS alone (35, 45, 46). Lesions that determine later neurodevelopmental outcome might be more microscopic and requiring specific imaging techniques. Pre-discharge cerebral CT/MRI is advised to cover this (2).

Nowadays, MRI, for its high sensitivity and specificity to detect stroke and white matter injuries, is considered the best imaging technique to detect brain abnormalities related to previous ECMO treatment (46, 47). It is contraindicated during ECMO, however, and can only be performed after decannulation and when the patient can be safely transported. Rollins and coworkers showed that 50% of neonates with a normal CUS had an abnormal MRI; non-hemorrhagic lesions were most often missed by CUS (46). A CT scan is recommended on suspicion of acute neurological cerebral complications. In the acute stage of cerebral ischemia, 53% of the patients have normal CT scans (48). Transport of the patient on ECMO for CT scanning can be challenging. Moreover, the radiation exposure is another disadvantage. Portable head CTs are available in some PICUs.

The effects on the brain of ligation of the right carotid artery and jugular vein in VA-ECMO have been reason for concern. Most studies did not find left-to-right hemisphere differences for cerebral lesions (32–35, 45, 49). Still, Mendoza and colleagues found that hemorrhages occurred more often in the left hemisphere but ischemic lesions more often in the right hemisphere (50). Hahn and colleagues found a predominance of non-hemorrhagic lesions in the right hemisphere (51).

The effect of ECMO technique, VA versus VV, on the incidence of cerebral lesions is still unknown. In a retrospective study, the incidence of severe intracranial hemorrhage in VA-ECMO treated newborns was higher than that in VV-ECMO treated newborns (29 vs. 7%) (52).

Cerebral injury might originate from combinations of pre-ECMO factors, patient-related factors, disease-related factors and even factors related to ther ECMO treatment itself. Possibly contributing factors are: acidosis, sepsis as primary diagnosis, coagulopathy, heparinization, thrombocytopenia, venous congestion due to jugular vein ligation, inflammatory response on ECMO, and carotid artery ligation (53). Cerebral autoregulation, which is the mechanism to maintain cerebral blood flow over a wide range of cerebral perfusion pressures, is disturbed in severely ill term infants and in newborn lambs after hypoxia and/or ECMO treatment (54, 55). The cerebral vasculature is therefore then highly vulnerable to blood pressure changes, with risk of ischemic complications from hypoperfusion and hemorrhagic lesions from hyperperfusion.

In a lamb model, cerebral blood flow was increased after hypoxia and oxygen transport to the brain, although cerebral oxygen metabolism was still maintained when the carotid artery and jugular vein were ligated (56). With near infrared spectrophotometry (NIRS) and Doppler ultrasound it could be demonstrated that carotid artery ligation caused a temporary decrease in cerebral oxygenation in both hemispheres (57, 58). Initiation of ECMO led to a significant increase in cerebral blood volume and cerebral blood flow velocity. All together these may be risk factors for cerebral hemorrhages during ECMO treatment. Furthermore, changes in ECMO flow during the ECMO run can alter regional cerebral autoregulation, as was shown in a small study using multichannel NIRS (12 channels) and wavelet cross correlation of oxygenated hemoglobin with mean arterial blood pressure (59).

### Seizures

Seizures are related to a worse outcome but are usually only registered during EEG (60). In particular in neonates and young children, seizures can be subtle or subclinical and only detectable by continuous EEG monitoring, which also gives information about the background pattern, organization, and inter-ictal burden (61–63). Changes in background pattern, in particular in one hemisphere, can be a symptom of acute brain damage. As a downside, prolonged EEG monitoring harms the patient's scalp, and many children develop edema during ECMO runs, which can affect the EEG signal. This is why trained EEG staff and neurologists should set up the montage and interpret the EEG findings. Studies on continuous EEG monitoring in ECMO patients have not been published.

Amplitude integrated EEG (aEEG), which compresses raw EEG information from 1-2 channels, is commonly used as an alternative for EEG–although it has a poor sensitivity in seizure detection (25–84%) (64). Focal seizures and neonatal seizures of very low frequency are likely be filtered out of the aEEG trace (64). In a study in 26 neonates treated with ECMO, subclinical seizures were detected in two cases; in seven cases (38%) the aEEG findings were classified as moderately to severely abnormal (65). In another aEEG study, in 20 ECMO-treated neonates, severe abnormalities detected before and/or during ECMO predicted death or moderate to severe intracranial neuropathology, as confirmed by neuroimaging or autopsy (66).

### Diagnostic tools to predict neurodevelopmental outcome

Irrespective of the neuroimaging technique used, conflicting results have been reported on the relation between imaging abnormalities and long-term neurodevelopmental outcome. Neonates with CUS abnormalities do not always develop neurological disabilities (46, 67). The other way round, a normal CUS does not guarantee normal neurodevelopment (35). The correlation between early neurodevelopmental outcomes and CT (37) or MRI (46) findings is poor. Still, neonates with increased cerebrospinal fluid spaces seem to be at risk for impaired neurodevelopmental outcome (68).

NIRS technology is a functional imaging technique that employs low-energy optical radiation (mostly in 2–3 different wavelengths) to assess absorption changes in the underlying brain tissue. These absorption changes reflect the changes in local concentration of oxy- and deoxy-hemoglobin, which in turn are related to and triggered by the alternation in neuronal activity. In a recent case series in 34 neonates and infants younger than 3 months, continuous NIRS monitoring during ECMO revealed reduced brain tissue oxygenation in non-survivors and patients who had neurological injury on neuroimaging compared to those with more favorable outcome. This observation suggests a potential role for NIRS in prognostication-in spite of the fact that the NIRS values can be influenced by several factors. The threshold value for brain ischemia still needs to be established (69).

## Long-term outcomes

The long-term medical and developmental outcomes after neonatal ECMO have recently been reviewed (70, 71). The highlights from this review and recent literature are addressed below. Note that such data are not available for ECMO for respiratory failure in older children and for cardiovascular disease requiring ECMO support (72, 73). The scarce data on neurodevelopmental follow-up in older children will be discussed below.

### Medical outcomes

#### Pulmonary morbidity and exercise tolerance

Studies on long-term lung function after neonatal ECMO are scarce. The findings can be summarized as follows. Mild airflow obstruction with normal lung function and normal diffusion capacity are mostly noted at follow up examinations (during school age) (70). The underlying diagnosis—i.e., respiratory distress syndrome or CDH—or the extent and duration of barotrauma and oxygen therapy are considered as risk factors for persisting respiratory morbidity (70, 74). Normal or decreased exercise tolerance is seen at school age (70). There are no comparable data on long-term pulmonary outcomes or exercise tolerance in children who underwent ECMO treatment after the neonatal period, due to many different underlying diagnoses and institutional ECMO protocols.

#### Renal function

Two-thirds of neonates receiving ECMO develop acute kidney injury (AKI) (75). In a study in 3,865 critically ill children aged 2 weeks to 18 years, those who needed ECMO treatment were at risk for developing AKI (adjusted odds ratio 2.72) (76). Long-term renal function testing after neonatal ECMO, both in children with and without AKI at a mean age of eight years, revealed at least one sign of chronic kidney disease in 54/169 (32%) of children (77).

### Neurodevelopmental outcomes

**Figure 1:**
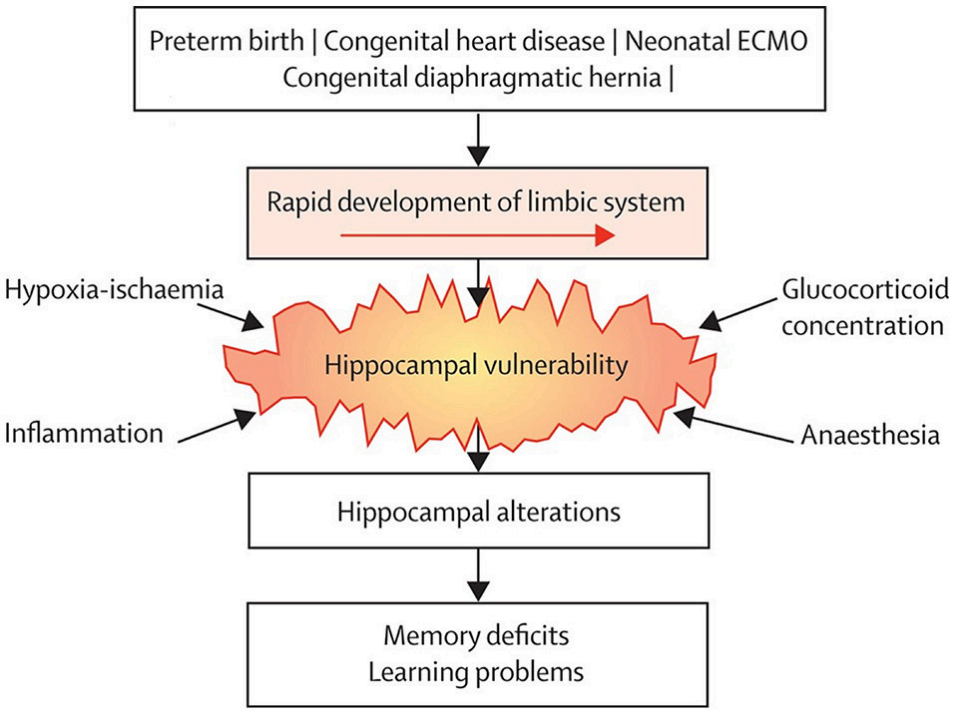
Common neurodevelopmental pathway following neonatal critical illness. Survivors of neonatal critical illness share an increased risk of hippocampal alterations due to vulnerability to common conditions associated with neonatal critical illness, which leads to long-term memory deficits. ECMO = extracorporeal membrane oxygenation. Reprinted from The Lancet Child Adolesc Health Schiller et al. (83). Copyright 2018, with permission from Elsevier.

In the past 10 years, it has become more and more apparent that growing up after treatment with neonatal ECMO might be associated with long-term neurodevelopmental consequences. Recent extensive neuropsychological assessment in school-age children treated with neonatal ECMO has led to the delineation of a specific neuropsychological profile in many of these patients. Although IQ is generally within the average range throughout development, specific sustained attention deficits and impaired verbal and visuospatial memory, both immediate and delayed recall, have been found at school-age (78–80). Similar outcomes were described in 17-year-old neonatal ECMO survivors, suggesting that these deficits persist into adolescence (81). These findings suggest a “growing into deficit” phenomenon (82), where subtle injuries in specific brain regions acquired in early life become evident only later in life, at the time when those brain regions are required for higher cognitive functioning (83) (Figure 1).

The use of advanced neuroimaging techniques has shed light on the underlying neurobiology of the long-term neuropsychological deficits following neonatal ECMO. Sophisticated neuroimaging methods to study the brain revealed no differences in cortical thickness and global brain volumes between neonatal ECMO survivors and healthy controls at school-age (84). Nevertheless, the ECMO survivors had significantly lower verbal memory than controls (85). It seems, therefore, that the underlying brain injury in ECMO survivors is more subtle and found in specific areas. In another study, structural MRI in 12-year-old children who had experienced neonatal hypoxia showed smaller hippocampal volume in the left and right hemispheres, adjusted for total brain volume, compared to healthy controls (86). These findings were replicated by our group in school-age children treated with ECMO following severe respiratory failure: their hippocampal volume specifically associated with verbal memory was smaller than that in healthy controls (87). Using diffusion tensor imaging (DTI), we further showed that the global white matter microstructure in the former patients was different from that in healthy controls, specifically the white matter tracts of the cingulum and parahippocampal part of the cingulum (87).

The regions found to be affected following neonatal ECMO in these studies are all part of the brain's limbic system, which rapidly develops from the third trimester of pregnancy throughout the neonatal period (88, 89). As such, these areas may be particularly vulnerable in critically ill newborns. In the studies mentioned above, only limited cognitive assessment was available. Therefore, the underlying neurobiological correlates of the observed sustained attention and visuospatial memory deficits, in addition to verbal memory deficits, following neonatal ECMO remained unknown. In a different cohort of school-age (8–12 years) survivors of neonatal ECMO following severe respiratory failure and CDH, DTI, and structural MRI were combined with elaborate neuropsychological assessment to assess whether the previously shown brain alterations were associated with the specific attention and memory deficits observed (80). Again, we found that smaller hippocampal volume in ECMO survivors was associated with worse verbal memory (80). Furthermore, lower global fractional anisotropy (FA), which is generally interpreted as reduced coherence of white matter fibers (90), was associated with worse sustained attention (80). Higher mean diffusivity, suggestive of decreased integrity in axonal membranes, packing, or myelin (90), in the parahippocampal part of the cingulum was associated with worse visuospatial memory (80).

Taken together, these findings indicate that neonatal ECMO is associated with specific neurobiological alterations in the neuropsychological profile. As these children seem to “grow into their deficits,” it is imperative that structured, problem-oriented neuropsychological follow up throughout childhood and into adolescence is indicated. This is underlined by the high number of neonatal ECMO survivors who need extra help in school, despite normal intellectual abilities (79).

Of eight available studies on long-term neurological outcome in children who needed ECMO for cardiac failure, only four actually performed age-appropriate neuropsychiatric tests (73). The proportion of children available for follow-up ranged from 26-51% (73) and the cohorts overlap in two of these studies (91, 92). In a Canadian study in four centers, 42 percent of the 98 children requiring VA-ECMO for cardiac disease were neonates and all 44 survivors without chromosomal abnormality were assessed at a mean age of 52 months. The mean IQ was 79.7, and 25% of children had an IQ below−2 SD (91). The same group reported on neurodevelopmental outcomes in a subset of 17 five-year-old ECPR survivors with cardiac disease who were assessed at least 6 months after ECPR. This subset had a mean IQ of 76.5, and 24% had an IQ below−2 SD (93). Wagner and coworkers evaluated 14 children who underwent ECMO (10 for cardiac disease and 4 for respiratory failure) after the first month of life at a mean age of 7.2 years. The total study cohort consisted of 22 participants (8 neonatal ECMO cases) born between 1991 and 2004 (94). The mean IQ of these 22 children was 74.7. In 2003, Hamrick and coworkers reported neurodevelopmental outcome at a median age of 55 months in 14 cardiac ECMO survivors. The median age at initiation of ECMO treatment was 27 days, which implies that only half of this cohort underwent ECMO beyond the neonatal period. Evaluation revealed normal cognition in 50% and normal neuromotor outcome in 72% (95).

## Current status of follow-up programs

The current recommendations for long-term follow-up published by ELSO have been established in 1994 and were reviewed in 1997 (96). Based on the knowledge available at that time, ELSO recommended to discontinue follow up after the age of 5 years and perform one assessment after 6 months in those who needed ECMO support during childhood.

Only very few ECMO centers in the world provide comprehensive multi-disciplinary follow up right from the time of discharge from hospital to adolescence. The 10 years' experience of a single center follow-up program in the UK for children supported for respiratory ECMO has recently been published (97). Follow-up was offered to all 194 survivors at the age of 1 year. Neurodevelopmental problems were identified in 30% of 98 participants, but alarmingly, 66 of 96 non-participants (34% of all ECMO survivors) were completely lost to follow-up and did not seem to attend local health care services (97). Most data on long-term outcomes after neonatal ECMO have become available from a nationwide longitudinal follow-up program in the Netherlands, which is offered as standard of care to all neonates and children discharged after ECMO treatment (98). Seventy-nine percent attended the follow-up program at 8 years (99) and 63% underwent medical and developmental evaluation including neuropsychological assessment at 17–18 years (81).

### Standardized clinical assessment and management pathway; the UK experience

In 2010, Rathod et al. proposed a novel methodology to aid the rationalization of clinical management and permit evolution of care pathways (100). This methodology is founded on the understanding that most clinical decisions are not necessarily evidence-based, and that there must be provision for flexibility in relation to changing practice. As part of the quality improvement project, an attempt was made to establish a collaborative Standardized Clinical Assessment and Management Pathway (SCAMP) for neurodevelopmental outcome between ECMO centers in the UK. In accordance with the difference steps in SCAMP, published literature, international recommendations and local guidelines were reviewed. Expertise from the fields of neurology, neuroradiology and neuropsychology was sought and a consensus was evolved through a series of local and national specialist group meetings. The aim of this initiative was to identify surveillance, screening and early interventions to improve the level of functional neurodevelopment, quality of life and family satisfaction in children post mechanical support. A framework for cyclical analysis was defined, allowing variability in outcomes to emerge alongside refinements in care and resource utilization. Although SCAMPs can potentially reduce resource utilization and costs, as recently evidenced for treatment with arterial switch operations (101), available resources are currently insufficient to guarantee neurodevelopmental assessments in the long run.

### The importance of age-appropriate follow-up programs

For clinicians it is important to know about long-term morbidities as more children with severe comorbidities will survive. They may, therefore, be confronted with problems never encountered in the past. Moreover, as functional outcomes may be related to treatment, evaluation of long-term effects of treatment interventions or after implementation of standardized postnatal treatment protocols is important.

**Figure 2:**
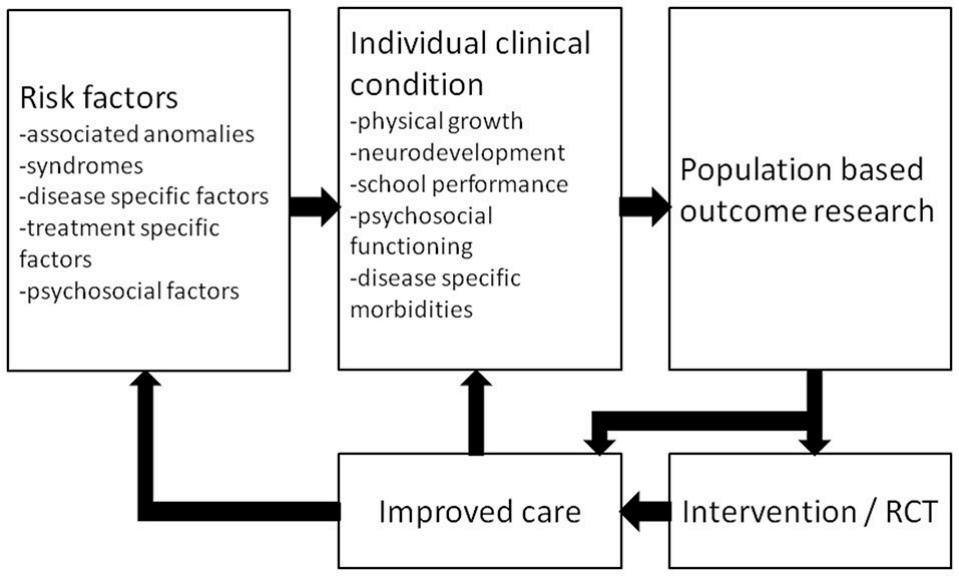
Schematic representation of a standardized multidisciplinary approach to optimize care. RCT = randomized controlled trial. Reprinted from Sem Pediatr Surg, Vol 26, IJsselstijn H et al., Assessment and significance of long-term outcomes in pediatric surgery, Pages 281–285, Copyright 2017, with permission from Elsevier.

From the patients' perspective, knowledge on long-term morbidities will help to recognize problems at an early stage so that timely intervention can be offered. For instance, a patient should be referred to a pediatric physical therapist if gross motor function problems that have implications for everyday activities persist. Explaining expected long-term outcomes to the child, its parents and other caregivers will have a stimulating effect on care domains such as self-management, family empowerment, and education (102). Awaiting further research, we hypothesize that long-term follow-up programs are cost-effective as family empowerment is expected to improve outcome and education of other caregivers may result in targeted evaluation without redundant tests. Moreover, outcome research data can form a basis for randomized clinical trials that lead to improved care (Figure 2). Such a randomized controlled trial, on the effectiveness of a working-memory training on improving long-term neuropsychological outcome following neonatal ECMO and/or CDH, showed sustained improvements in visuospatial memory in school-age children trained with Cogmed Working-Memory Training (103).

### Recommendations for age-appropriate follow-up

Recommendations for long-term follow up depend on the presence of neurological comorbidity, the nature and extent of the underlying disease, and the indication for ECMO. In any case it should preferably be offered as standard of care, and in a structured and standardized way. This permits to evaluate outcome data and effects of interventions. Such a program would preferably be offered within the ECMO center, but could also be provided by community pediatricians and other health care providers near home. As neurodevelopment is usually normal within the first years of life, health care providers and parents may be inclined to disregard the necessity of regular follow-up visits. Based on current knowledge on long-term outcomes and the phenomenon that survivors of ECMO may “grow into their deficits,” health care providers working in ECMO centers should inform parents about potential sequelae and urge them to seek advice in case of unexplained growth failure, reduced exercise tolerance or neurodevelopmental problems such as clumsiness, failure of academic performance, behavioral problems or impaired attention, concentration, or memory.

**Figure 3:**
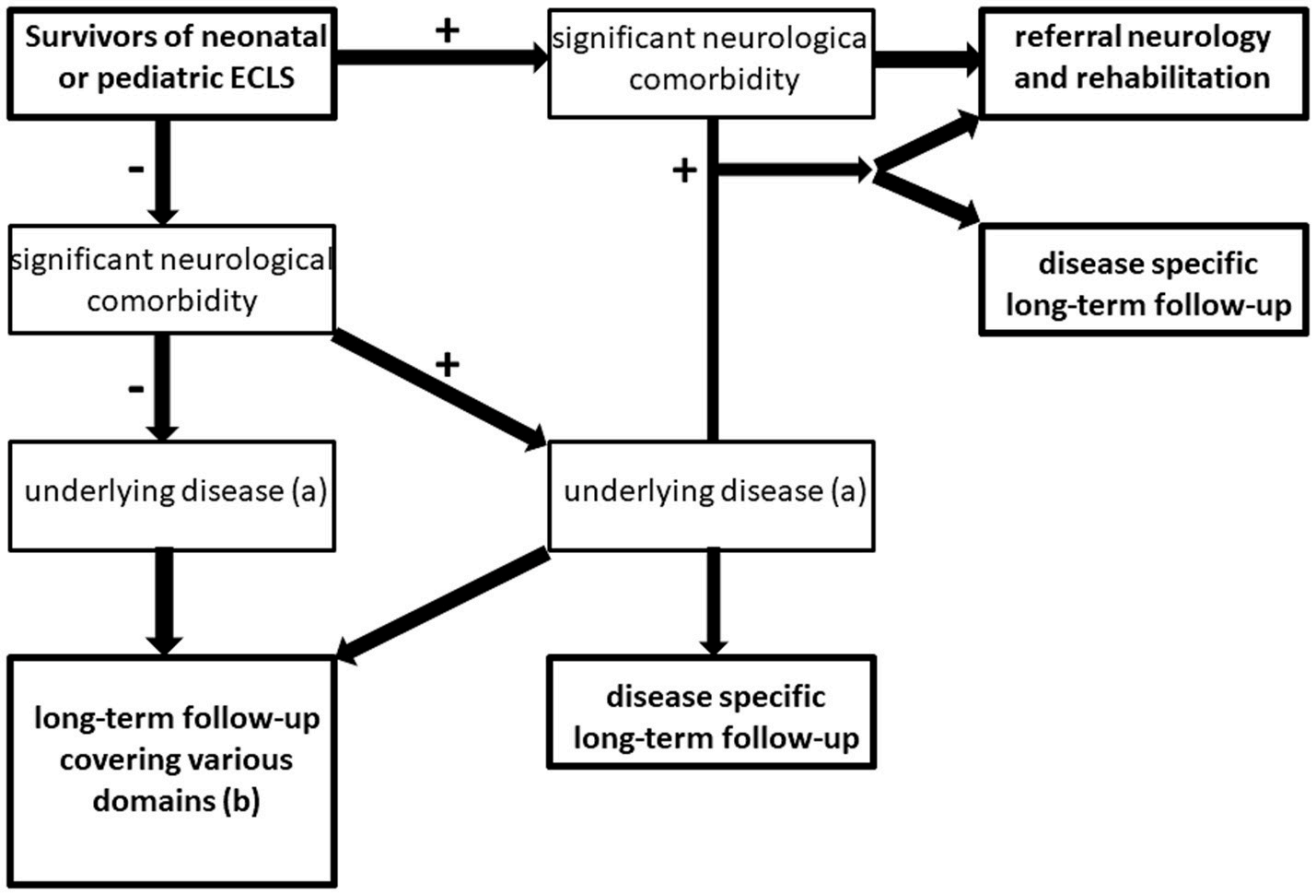
Flowchart for long-term follow-up of ECMO survivors. +: indicates condition present; – indicates condition absent, (a) e.g., cardiovascular disease, pulmonary disease, genetic syndrome, psychiatric disease (including delirium, post-traumatic stress disorder or anxiety disorder following critical illness) (b) see Table 1 for domains that should be covered in follow-up of ECMO survivors without (serious) neurologic comorbidity.

We propose the following flowchart for follow-up as standard of care (Figure 3).

Children *with* neurological comorbidity should be referred to a neurologist and/or a rehabilitation center. Also in case of suspected neurological comorbidity that is not evident at discharge, referral to a pediatric neurologist and/or a developmental pediatrician is recommended. Moreover, if children suffer from underlying disease (e.g. cardiovascular disease or lung function impairment) additional disease-specific follow-up should be arranged.

**Table 1:** Proposal for and relevance of long-term follow-up after (neonatal) ECMO.

	**Assessments**	**Domains of interest**	**Relevance/intervention**
**INFANCY**
0–2 years	Growth Kidney function Hearing assessment Neurologic assessment including imaging Mental development Motor development	Hypertension, urinary protein-to-creatinine ratio MRI brain	Referral dietician Referral nephrologist (CKD) Early referral audiology Early recognition, rehabilitation Early referral Referral physical therapist
**PRESCHOOL AGE**
2–5 years	Growth (mainly CDH) Kidney function Neurologic assessment Language development Motor development	Hypertension, urinary protein-to-creatinine ratio	Referral dietician Referral nephrologist (CKD) Rehabilitation Hearing assessment, referral speech-language pathologist Referral physical therapist
**SCHOOL AGE**
≥6 years	Growth (mainly CDH) Kidney function Lung function assessment Motor development Exercise capacity Neuropsychological assessment Behavior assessment	Hypertension, urinary protein-to-creatinine ratio Spirometry Intelligence (only once in follow-up) Memory Attention/concentration/information-processing Hyperactivity Somatic problems	Referral dietician Referral nephrologist (CKD) Evaluate reversibility of airflow obstruction Referral physical therapist Sports participation and/or exercise training Referral to early school support Referral to cognitive rehabilitation Referral to support/guidance
**ADOLESCENCE**
>12 years	Growth (mainly CDH) Kidney function Motor function Exercise capacity Neuropsychological assessment Behavior assessment	Hypertension, urinary protein-to-creatinine ratio Gross motor function (e.g., ball skills) Memory Attention/concentration/information processing Hyperactivity Depressed feelings/social problems Somatic problems	Referral dietician Referral nephrologist (CKD) Referral physical therapist/sports participation Sports participation/exercise training Referral to school support Career support/choice of profession Referral to cognitive rehabilitation Referral to support/guidance

*ECMO, extracorporeal membrane oxygenation; CKD, chronic kidney disease; CDH, congenital diaphragmatic hernia*.

The majority of children (i.e., up to 85–90%) may be discharged from the ICU without neurological comorbidity. Children *without* neurological comorbidity at discharge should be referred for disease-specific follow-up if applicable. Both for children with or without underlying disease (e.g., respiratory neonatal ECMO for MAS), a long-term follow-up program with regular assessments covering various medical and neurodevelopmental domains is recommended (Table 1).

From the current literature it is unknown whether children who need ECMO at older age have similar long-term problems as neonatal ECMO survivors. As somatic problems may occur at any age and brain development including myelinization continues up till adolescence, it can be assumed that also pediatric ECMO survivors benefit from long-term follow-up and timely interventions.

## Risk stratification

Interestingly, the neurodevelopmental outcomes in patients treated with neonatal ECMO following severe respiratory failure resemble those of patients treated with conventional management (86), and do not differ between children with congenital diaphragmatic hernia treated with and without ECMO (78, 80, 104). These findings suggest that, rather than treatment or underlying disease, similar factors associated with critical illness may be involved in the long-term sequelae (83). Risk stratification should therefore not only focus on the ECMO treatment and post-discharge assessments but also take into account the underlying pathology, disease severity and treatment-related morbidities such as infections and coagulation problems. The latter morbidities are considered as iatrogenic risk factors that sometimes significantly affect overall outcome. Different options that may be helpful to risk stratification are discussed below.

### Registries

In pediatric critical care medicine, many registries collect data of variable levels of clinical detail–ranging from vital signs and laboratory results (high clinical detail) to demographic and administrative data (low clinical detail) (105). To date, several large, multi-center prospective registry datasets provide important clinical information about a population that is heterogeneous with respect to age and comorbidities at PICU admission (106). Apart from these registries and disease-specific registries, therapy-specific registries are available, such as the ELSO Registry (105). The ELSO registry was set up to provide member institutions with data to benchmark their results against that of other institutions, to improve quality of care to patients, and to achieve cost-effectiveness. Registered data include sex, ethnicity, nature and severity of illness, technical details of extracorporeal support used, complications and outcome^1^ Combining data from the large pediatric critical care datasets, the disease-specific registries, the ELSO Registry and long-term outcome data would be very helpful to determine which children are at risk for long-term morbidities and personalized care pathways can be offered at PICU discharge. However, we have to realize that registries are generally based on voluntary data provision without standardized quality control, which may influence the results.

### Neuropsychological assessment and neurobiological correlates

Children at risk of neuropsychological impairments and academic problems following neonatal ECMO should be identified as early as possible and supported. Neuropsychological assessment is needed to identify children at risk of school problems. Since higher-order cognitive functions, such as memory, are still developing throughout early childhood, results of such assessments cannot be interpreted reliably until school age (107). In practice, children who already experience problems are offered neuropsychological assessment, which thus is a diagnostic tool rather than a prediction tool. It would be valuable to find predictors for later school problems that can be assessed as early as in infancy.

Promising in this respect is MRI scanning, which is non-invasive and can be reliably performed in infants without sedation (108, 109). Hippocampal volume alterations detected in infancy with MRI could potentially serve to predict memory deficits later in childhood. For instance, hippocampal volumes in preterm infants measured at term-equivalent age were found to correlate with memory outcomes both at 2 years and seven years of age (108, 109). Future studies aimed at finding normative infant hippocampal volumes are a first step toward early risk stratification using MRI in critically ill infants. Furthermore, as the hippocampus is rapidly growing during the first two years of life (110), longitudinal studies will determine the optimum timing to assess hippocampal morphology. Longitudinal studies can provide more insight in the association between neurodevelopmental outcome and clinical characteristics, and contribute to early risk stratification. As a complex interplay among different factors associated with the underlying disease, pharmacological and non-pharmacological treatment and “iatrogenesis” is likely to determine a child's neurodevelopmental outcome (83), detailed clinical information should be collected during the perinatal period.

## Concluding remarks and additional recommendations

Since the introduction of extracorporeal life support several decades ago (111), the population, devices and techniques have changed and more children survive. Interest has therefore shifted from reduction of mortality toward prevention of morbidity. Nowadays, more and more clinicians become aware that multidisciplinary long-term follow up should be offered to ECMO survivors.

To optimize care as suggested in Figure 2, several issues need to be resolved on the way to uniform data collection in a multicenter international registry of long-term outcome data. First, treatment protocols, including recommendations for neuromonitoring in the ICU, need further standardization. Although registered long-term outcome data may be useful to compare different treatment strategies, a minimum set of uniform treatment criteria, baseline data, and a substantial number of participating centers are required for the detection of statistically significant differences. Second, one uniform follow-up program should be applied. Frequent revision of the follow-up guidelines—accounting for new treatment modalities—is an important first step. Moreover, assessment instruments and outcome scores need to be standardized and validated and population-specific standard deviation scores should be obtained. Third, health care policy makers need to realize that sufficient resources must be ensured, not only to make long-term follow-up possible, but also to set up registries meeting the institutional criteria of data management for all participants and to maintain registries both at a local and a central level. Organizations such as ELSO and international societies of pediatric and neonatal intensive care should have an important role in facilitating multicenter collaboration and to support initiatives to obtain sufficient funding.

## Author contributions

HI, MH, RS, RH, AH, and AvH: drafted the manuscript. All authors contributed to critical revision of the manuscript and approved the final version before submission.

### Conflict of interest statement

The authors declare that the research was conducted in the absence of any commercial or financial relationships that could be construed as a potential conflict of interest. The reviewer RS and handling Editor declared their shared affiliation.

## Footnotes

^1^ELSO Data Policy. Available online at: https://www.elso.org/Registry/DataRequest.aspx.
